# Treatment of Chagas Cardiomyopathy

**DOI:** 10.1155/2013/849504

**Published:** 2013-11-24

**Authors:** Fernando A. Botoni, Antonio Luiz P. Ribeiro, Carolina Coimbra Marinho, Marcia Maria Oliveira Lima, Maria do Carmo Pereira Nunes, Manoel Otávio C. Rocha

**Affiliations:** ^1^Postgraduate Course of Infectious Diseases and Tropical Medicine, School of Medicine, Universidade Federal de Minas Gerais, 30130 100 Belo Horizonte, MG, Brazil; ^2^Hospital das Clínicas, Universidade Federal de Minas Gerais, 30130 100 Belo Horizonte, MG, Brazil; ^3^Fundação Hospitalar do Estado de Minas Gerais, 30150 260 Belo Horizonte, MG, Brazil; ^4^School of Medicine, Universidade Federal de Ouro Preto, 35400 000 Ouro Preto, MG, Brazil; ^5^Departamento de Fisioterapia, Universidade Federal do Vale do Mucuri e Jequitinhonha, 39100 000 Diamantina, MG, Brazil

## Abstract

Chagas' disease (ChD), caused by the protozoa *Trypanosoma cruzi* (*T. cruzi*), was discovered and described by the Brazilian physician Carlos Chagas in 1909. After a century of original description, trypanosomiasis still brings much misery to humanity and is classified as a neglected tropical disease prevalent in underdeveloped countries, particularly in South America. It is an increasing worldwide problem due to the number of cases in endemic areas and the migration of infected subjects to more developed regions, mainly North America and Europe. Despite its importance, chronic chagas cardiomyopathy (CCC) pathophysiology is yet poorly understood, and independently of its social, clinical, and epidemiological importance, the therapeutic approach of CCC is still transposed from the knowledge acquired from other cardiomyopathies. Therefore, the objective of this review is to describe the treatment of Chagas cardiomyopathy with emphasis on its peculiarities.

## 1. Introduction

Chagas' disease (ChD), caused by the protozoa *Trypanosoma cruzi *(*T. cruzi*), was discovered and described by the Brazilian physician Carlos Chagas in 1909. The infection is spread by the passage of the protozoa from the infected faeces of blood sucking insects through the skin at bite sites or the mucosa, through blood transfusion or transplantation, or orally through contaminated food. After a century of original description, trypanosomiasis still brings much misery to humanity and is classified as a neglected tropical disease prevalent in underdeveloped countries, particularly in South America [1–3]. It is now an increasing worldwide problem due to the number of cases in endemic areas and the migration of infected subjects to more developed regions, mainly North America and Europe, where there are more than 100,000 people who might potentially transmit the disease by either hemotransfusion, or organ donation, or pregnancy [4, 5].

## 2. Epidemiology

Worldwide, 10 million people are estimated to be infected with *T. cruzi* and more than 100 million people are at risk of infection. The incidence is around 56,000 cases per year [1, 2]. Amongst those infected, about 20–40% have clinical features of some cardiac injury (chronic chagas cardiomyopathy, CCC), and 15% ultimately develop overt heart failure due to left ventricular dysfunction [5–9].

Chagas' disease occurs mainly in Latin America and it is the major cause of disability among young adults secondary to tropical diseases in the region [8]. Estimated annual deaths globally decreased from 45,000 in 1990 to around 11,000 in 2008. Annual incidence during this 16-year period fell from 700,000 to 56,000. The burden of Chagas disease has been reduced from 2.8 million disability-adjusted life years to less than 500,000, and 750,000 productive life years are lost [1, 2]. The cost of care and prevention is US$ 1,200 million per year [6, 8]. Control of the vector was achieved in countries like Brazil, Uruguay, Argentina, and Chile two decades ago, but in others, all phases of the disease are still observed. However, unfortunately, recent reports demonstrate that Chagas' disease has reemerged where control had once been successful, in regions such as the Chaco region of Argentina and Bolivia. Beyond the endemic areas, Chagas disease also represents worldwide public health problem due to migration of infected people to developed countries, mainly North America and Europe [1, 2, 6].

## 3. Clinical Presentation and Pathology

The disease characteristically presents two phases, acute and chronic, each with distinct clinical features. The acute phase usually presents with nonspecific symptoms accompanied by intense protozoan invasion of many organs and tissues. After the specific immunologic response against *T. cruzi*, parasitism decreases and the chronic phase starts. Histopathological studies of the acute phase of the Chagas disease show intense parasitism associated with diffuse inflammation, whereas in the chronic phase, the low levels of parasite antigen detected by PCR and intense cell destruction are disproportional [9, 12]. Two forms are typical in the chronic phase. The indeterminate form, which represents about 60–70% of the cases, is diagnosed when there are positive serologic test results, but no specific organic injury of the oesophagus, bowels, and/or heart is detected [6]. Among patients with the indeterminate form, nearly 2–5% per year evolve to determinate forms, which are generally mild [6]. In the determinate forms, cardiac and digestive involvement are the main features and cardiac injury is the main prognostic determinant of the disease [6]. 

Chronic Chagas cardiomyopathy is the most important chronic form of Chagas' disease due to its high morbidity and mortality and its significant medical and social impact. It is clinically classified according to the symptoms, electrocardiograph and radiological abnormalities, and changes in left ventricular function (Table 1). Electrocardiography abnormalities, male gender, systolic blood pressure less than 120 mmHg, impaired systolic function, left ventricular dilatation, VO_2_max, and the occurrence of complex arrhythmias during exercise testing predict a poor outcome. The most important prognostic marker is the intensity of myocardial dysfunction [6, 7, 12]. Risk scores were developed to stratify the 5- and 10-year risks of death [13, 14], although their clinical utility is still uncertain. A systematic review of observational studies about predictors of mortality in chronic Chagas' disease concluded that impaired left ventricular function, NYHA class III/IV, cardiomegaly, and nonsustained ventricular tachycardia (NSVT) are the main markers of a poor prognosis [14]. 

Despite its importance, CCC pathophysiology is still poorly understood. Although cell destruction by the parasite is significant in the acute phase, it does not seem to be the main mechanism in the chronic phase since parasitism is scarce and disproportionately less intense than tissue lesion and large inflammation [9]. The paradox of severe heart involvement and little Chagasic antigen has prompted many researchers to propose autoimmune phenomena as an important mechanism in the pathogenesis of Chagas cardiomyopathy [9, 12]. Other important mechanisms are immune-mediated cell destruction, microvascular abnormalities, and degeneration of nerve endings [9, 12]. Chagas cardiomyopathy does not seem to differ from other cardiomyopathies with regard to hemodynamic, neurohormonal, and inflammatory responses. This common pathophysiology suggests that treatments effective in classic heart failure trials should be beneficial in CCC. However, CCC has several peculiarities, such as frequent ventricular arrhythmias, and several forms and grades of conduction disturbances, such as sinus bradycardia, complete atrioventricular block, and right bundle block [6, 7, 13, 15–17]. Morphologically, hypertrophy, dilatation, and severe fibrosis are prominent and in up to 40% of the cases, and an apical ventricular aneurysm is present [3, 4]. One group argues that Chagas cardiomyopathy gives rise to the most intense fibrous reaction amongst all causes of myocarditis, leading to severe disorganisation of the myocardial architecture and structure [5, 18]. These combined peculiarities lead to high incidences of sudden death (60% of all deaths), cardiac insufficiency, and ventricular remodelling [19]. 

## 4. Treatment

Independently of its social, clinical, and epidemiological importance, the therapeutic approach of CCC is still transposed from the knowledge acquired from other cardiomyopathies. Studies forwarded to chagasic patients are few and samples are generally small or experimental animals.

Roberti et al. divided 17 NYHA IV chagasic patients into two groups and administered either captopril or placebo. Smaller cardiac frequency, fewer ventricular arrhythmias, and significant decrease in urinary norepinephrine levels were observed in the captopril group. Both groups received digoxin and furosemide [20]. Khoury et al. observed neurohormonal control in chagasic patients after the use of digoxin plus enalapril [21]. Freitas et al. evaluated the effects of *β*-blocker in chagasic patients and observed greater improvement in those with lower cardiac frequency than otherwise [22]. Davila et al. (2002) demonstrated a decrease in left ventricular systolic diameter and an increase in LVEF in a NYHA III/IV CCC group followed up after the administration of metoprolol [23]. Carvedilol was used safely in a chagasic group in the Acordes Trial Investigators and contributed to the improvement of LVEF [24]. 

The perception that the response of chagasic patients to the drugs usually prescribed in heart failure may be different [19] has led to suboptimal dosing or lack of initiation of medications with proven efficacy in patients with heart failure of other etiologies. Many physicians believe that patients with Chagas cardiomyopathy do not tolerate high doses of angiotensine-converting enzyme inhibitors (ACEI) [19]. Additionally, beta blockers are often not used due to presence of frequent bradycardia and conduction disturbances. Our group published [25] a randomised, placebo-controlled trial with ACEI and spironolactone followed by a *β*-blocker which showed that such treatment is safe, haemodynamically well tolerated, and not associated with symptomatic bradycardia. Significant improvement was observed, especially after the optimisation of the use of inhibitors of the renin-angiotensin system [25]. However, preliminary results of a study of CCC patients with moderate-to-severe CHF by Bestetti et al. showed that only 37% of the patients tolerated carvedilol after the inhibition of the renin-angiotensin system with high doses of ACEI [26]. Notwithstanding, recent findings by Issa et al. challenge current conservative practices in prescribing beta blockers to patients with heart failure secondary to Chagas' disease. In a subanalysis of a prospective trial on the effects of repetitive educational programs for adherence in heart failure treatment, the REMADHE trial [27], the authors reported lower frequencies of death or heart transplantation among patients with Chagas' disease under beta blocker therapy compared to those not receiving beta blocker (*P* < 0.001) [28]. Despite being a major cause of HF in Latin America, patients with ChD and HF were not included in large trials that validated the use of these drugs. The effectiveness, efficacy, safety, and tolerability of these drugs in patients with CCC have been based on these small studies and its use is extrapolated due to the benefit obtained in HF from other causes. In this way, the treatment should be based on the use of a combination of the following drugs: diuretics, angiotensin-converting enzyme inhibitors or angiotensin receptor blockers, aldosterone inhibitors, and beta blockers. Therefore, it has been suggested that the treatment should be in accordance to general guidelines for HF treatment, starting with ACEi (or ARB), aldosterone inhibitor, and diuretics, and additionally, beta blockers after clinical optimization (Table 2).

With regard to the thromboembolic events, these are a very important problem in ChD patients leading to great disability and mortality. There are doubts whether these patients have an inherent coagulation disturbance or not. Pinazo et al. designed a study to determine whether a prothrombotic state existed in chronic *Trypanosoma cruzi*-infected patients. They found that endogenous thrombin potential (ETP) and prothrombin fragment 1 + 2 (*F*_1+2_) showed values outside the normal levels in patients compared with controls [29]. Annually, the incidence of thromboembolic events is between 1 and 2% in patients with CCC, and the majority of cases occur in the subgroup of patients with HF. In this series, apical aneurysm and LV mural thrombosis were observed in 23% and 37% of patients, respectively [30]. Prevention and treatment of thromboembolism in patients with CCC should be guided by standard clinical recommendations. Thus, anticoagulation should be considered in patients with atrial fibrillation, previous embolic events, mural thrombus, IPEC/FIOCRUZ score ≥ 4 and possibly in those with an apical aneurism. The role of antiplatelet drugs in the prevention of thromboembolic events has yet to be determined in patients with ChD [6] (Table 2).

CCC has an essentially arrhythmogenic nature characterized by highly dense and complex ventricular arrhythmias [28], which leads to the assumption that ventricular fibrillation constitutes the terminal event in most cases of sudden death, which accounts for most of the deaths in Chagas' disease [30, 31]. However, the prophylactic or therapeutic use of antiarrhythmic drugs in CCC remains controversial. Chiale et al. [31], Haedo et al. [32], and Rosenbaum et al. [33] showed that amiodarone is the most effective of the antiarrhythmic agents in patients with CCC. Scanavacca and Sosa [34] observed that there was a low risk of arrhythmia or death when the LVEF was above 30%, but there was a 100% recurrence rate and 80% mortality if patients had a functional class III-IV (NYHA) with an LVEF less than 30%. Rassi Jr. et al. consider that the administration of amiodarone to chagasic patients with complex ventricular arrhythmias is justified, particularly when associated with ventricular dysfunction [30]. Recently, the presence of NSVT on Holter monitoring was identified as an independent prognostic factor in chagasic patients [14, 16]. When present, NSVT was associated with a 2.15-fold increased risk of mortality [16]. Remarkably, the combination of NSVT and LV dysfunction was associated with a 15.1-fold increased risk of subsequent death compared to the risk of patients without either risk markers [16]. On the other hand, Sternick et al. reported cases of sudden death in CCC patients with LVEF larger than 45% [35]. Regarding the role of the implantable cardioverter defibrillators (ICDs) in patients with CCC, Martinelli et al. [36] studied a cohort that included 116 consecutive patients with ChD and an ICD implanted for secondary prevention. The aim of the study was to assess, during long-term followup, the ICD efficacy of a ChD considering all-cause mortality and appropriate ICD shock therapy rates. In conclusion, in a long-term followup, ICD efficacy for secondary sudden cardiac death prevention in patients with ChD was marked by a favorable annual rate of all-cause mortality (7.1%); 50% of the cohort received appropriate shock therapy. Recently Barbosa et al. [37], studying a cohort with 135 chagasic and nonchagasic patients, for a period of 266 days, have targeted primarily appropriate therapy (appropriate shocks or antitachycardia pacing) and secondarily the event-free survival defined as absence of death or appropriate therapy. In conclusion, they demonstrated a higher frequency of appropriate ICD therapy and a shorter event-free survival in chagasic than in nonchagasic patients. Cardinalli-Neto et al. studied 90 CCC patients with implanted cardioverter defibrillator (ICD) to determine predictors of all-cause mortality. All patients received amiodarone. They concluded that the number of shocks per patient per 30 days predicts the outcome in CCC patients treated with ICD [38, 39]. In summary, the use of ICDs is beneficial in patients with malignant, sustained ventricular tachycardia or those resuscitated from sudden cardiac arrest, especially with a reduced left ventricular ejection fraction. However, the antiarrhythmic treatment of CCC is still controversial and a conclusive guideline is necessary due to the importance of arrhythmias and sudden death in the outcome of Chagas' disease (Table 2).

The electrophysiologic test can be used to identify therapeutic strategies and to stratify the risk of patients with VT associated with CCC. Leite et al. [40] assessed the role of electrophysiologic testing to identify therapeutic strategies for the treatment of patients with sustained ventricular tachycardia and chronic chagasic cardiomyopathy treated with amiodarone or sotalol. The conclusion was that in patients with chagasic cardiomyopathy and sustained VT, electrophysiologic testing can predict long-term efficacy of Class III antiarrhythmic drugs [40]. In addition, substrate mapping of epicardial and endocardial surfaces of the left ventricle and radiofrequency ablation for VT in Chagas cardiomyopathy were tested and found to be acutely effective in preventing VT recurrences and appropriate ICD therapies in a recent small trial [41]. Therefore, based on these results, the electrophysiologic test seems to be justified as primary prevention in patients with CCC (Table 2).

On the other hand, the principal causes of symptomatic bradyarrhythmias are advanced atrioventricular block and sinus node syndrome, whose indications for implantation of a permanent pacemaker should follow current recommendations for other cardiomyopathies [30]. 

Although resynchronization therapy has become an established recommendation for patients with moderate-to-severe heart failure according to recent guidelines, very few patients with CCC were included in controlled randomized trials [42] and the clinical indication is controversial. 

A pathogenetic role has been attributed to a variety of autoantibodies against cardiac proteins in dilated cardiomyopathy [43, 44]. Antibodies against the C-terminal region from *T. cruzi *ribosomal P proteins were demonstrated to physically interact, with an agonist effect, with human second loop of G-protein-coupled receptors. Such autoantibodies (AABs) directed to *β*1 adrenergic autoantibodies (*β*1-AABs) and muscarinergic 2 autoantibodies (M2-AABs) are thought to be involved in the pathogenesis of CCC [45]. Therefore, considering the increasing acceptance of an autoimmune background in the pathogenesis of symptomatic ChD, new treatment regimens directed to the removal or specific inhibition of AABs, which are similar to those used in other diseases with autoimmune background, could become increasingly important. From this background, the elimination of pathogenetic AABs, such as the pathogenic *β*1-AABs, via any apheresis techniques could be suggested and promising for patients with CCC [46]. The idea of using apheresis in the treatment of CCC is supported by different methods of immunoadsorption (IA) therapy that have shown to result in hemodynamic benefit for patients with dilated cardiomyopathy (DCM) and severely impaired systolic function in previous small trials [43, 44]. The clinical scenario in which IA therapy will be most beneficial is still to be defined. Recently, results were reported from the introduction of aptamers as binders in the apheresis technique in order to clear plasma from the *β*1-AABs found in [47]. Oligonucleotide aptamers are new biotechnological tools that were reported for the first time in the early 1990s. These aptamers are short single-stranded or double-stranded RNA or DNA sequences that show a high affinity for their targets, binding and neutralizing diverse molecule species, including antibodies. Thus, aptamers could be a new strategy in the neutralization of *β*1-AABs of CCC patients, maybe representing the future in the cardiomyopathy treatment [46] (Table 2).

Cardiac tissue repair with cell therapy now presents as a promising therapeutic alternative for chronic Chagas cardiomyopathy [48, 49]. A phase II trial elicited the safety profile of autologous bone marrow cell transplantation in patients with end-stage congestive heart failure secondary to Chagas' disease [50]. In this trial, a small, but significant increase in ejection fraction could be detected, without increase in the frequency of arrhythmias or troponin I levels [50]. After these promising results, a multicentre trial was designed to test the efficacy of intracoronary delivery of bone marrow-derived mononuclear cells (BMNCs) in a cardiopathies study [51]. Ribeiro dos Santos et al. [52] recently published the result of the Chagas arm of the multicenter randomized trial of cell therapy in the cardiopathies study. A group of Chagas patients were submitted to cell therapy with autologous BMNCs. They concluded that intracoronary injection of autologous BMNCs does not improve left ventricular function or quality of life in patients with chronic chagasic cardiomyopathy.

However, there is still evidence that not all the beneficial effects of cell therapy are due to the transplanted cell themselves, but rather to paracrine effects, such as recruitment of circulating stem cells, or activation of resident cardiac cells [48]. Thus, another attractive therapeutic possibility would be the direct intervention in the mechanisms of parasite-triggered inflammation, through antagonism of cell adhesion and migration. Cell adhesion molecules have been successfully used in the treatment of chronic inflammatory diseases. In Chagas' disease, preliminary experimental studies on the use of chemokines resulted in a better control of parasitism, promotion of a protective immune response, and reduction of cardiomyocyte lesion formation [53, 54].

Heart transplantation (HTx) in CCC has crossed for three phases. In the first phase, Chagas' disease was an absolute contraindication. The second phase started when an adjustment was made in the immunosuppression protocol, with a lower dosage being adopted to avoid adverse effects (neoplasias and reactivation). Nowadays, the third phase, cardiac transplantation for Chagas' disease is a reality. CCC is the third most important cause of indication for HTx in Brazil [10]. Indications for HTx in ChD are not different from other HF causes. Registry of patients undergoing HTx suggests that the prognosis of recipients with CCC can be better than that observed in nonchagasic recipients. HTx has been associated with a similar incidence of rejection episodes in Chagas and non-Chagas recipients and also a lower incidence of infection episodes has been observed in Chagas in comparison to non-Chagas recipients. *T. cruzi* infection reactivation can be easily treated with specific drugs (benznidazole) and implies a very low mortality rate. Survival rates for Chagas recipients at 1 month, 1 year, 4 years, and 10 years followup are 83%, 71%, 57%, and 46%, respectively. This result is better than that seen in non-Chagas recipients [55]. The use of left ventricular circulatory support as bridge to heart transplantation as well as destination therapy or bridging to recovery has been considered as a valuable treatment option for patients with CCC who evolve with decompensated heart failure or cardiogenic shock. The indications are similar to those observed in other HF causes [56] (Table 2).

In relation to nonpharmacological interventions, the effects of exercise training in chronic heart failure are well established, particularly with regard to improved functional capacity. However, it has not been studied in Chagas cardiomyopathy yet. Recently, Lima et al. demonstrated the effects of exercise training on functional capacity and health-related quality of life (HQoL) in 40 patients with CCC. They concluded that in patients with CCC, exercise training was associated with a major improvement in functional capacity and HQoL, without any adverse effects during the exercise protocol [57]. Previous studies have demonstrated that right ventricular function is a maker of diastolic function and was determinant to functional capacity in individuals with CCC [58, 59].

Specific treatment targeted at *T. cruzi* is effective in the acute phase with a chance of cure of nearly 90%; however, results in the chronic phase are indeterminate [60]. There are two drugs available: nifurtimox (Lampit) and benznidazole (Rochagan). Neither drug is approved by Food and Drug Administration, but both are available under investigational use protocols (Centers for Disease Control and Prevention Drug Service). These drugs have variable efficacy and should be taken for extended periods, and patients may experience severe side effects. The treatment of acute and congenital infection is most effective. Although there are no large-scale studies to support treatment efficacy, parasitological cure is believed to occur in 60% to 85% of persons with acute infection who complete a full course of either drug. In congenitally infected infants, the rate of parasitological cure has been shown to be >90%, if the treatment is given during the first year of life [11, 60]. Recommendations suggest that immediate treatment should be provided for all acute cases caused by vector transmission, oral transmission, congenital infection, laboratory accidents, organ transplantation, or any other route and for cases in the recent indeterminate chronic phase. Despite the symptoms, acute cases are considered to be present if in the acute or initial phase when *T. cruzi* is found in peripheral blood, as determined, mainly, by direct microscopic examination of fresh blood smears or by concentration methods (e.g., microhematocrit, Strout, or QBC). Treatment of the indeterminate phase has been established in children up to 12 years of age based on the positive results obtained from specific treatment by Andrade et al. [61] in Brazil and Sosa-Estani et al. [62] in Argentina. Cases of the later chronic form in children over the age of 12 could be treated but poor results have been obtained with regard cure; however, these results were not considered in relation to evolution of the disease [60] (Table 2).

Regarding etiologic treatment of the chronic phase of Chagas' disease, many questions remain unanswered, because there are no convincing studies with sufficiently large samples and adequate control groups that may indicate whether a specific treatment is effective in preventing evolution of the chronic phase of Chagas' disease [60]. Based on intense inflammatory reactions observed in histologic studies, and in secondary autoimmunity mechanisms, there is now consensus that these mechanisms always result from the presence of *T. cruzi* and its antigens. Accordingly, reductions in parasite and/or antigen burden, or its complete elimination, could help to prevent the formation of new inflammatory foci and the extension of tissue damage, thus promoting tissue restoration [60]. Based on these hypotheses, a large randomised, double-blind, controlled study (BENEFIT Trial) with benznidazole versus placebo is currently being performed in order to address this therapeutic dilemma [63] (Table 2).

Chagas cardiomyopathy remains largely neglected despite its medical and social relevance; multicentric, large and well-conducted randomised trials with CCC patients should be an international priority. Much more attention and specific research strategies and resources are necessary to reduce morbidity and mortality and to improve the quality of life of many people in Latin America and in other regions of the world. 

## Figures and Tables

**Table 1:** Clinical Classification of Chronic Chagas Cardiomyopathy [10].

Clinical group	Characterization
Stage A Chronic indeterminate Form	Asymptomatic, no significant alteration on physical examination, ECG, chest X-ray, esophagogram, and barium enema. No change on evaluation by sensitive techniques (echocardiogram, exercise testing, Holter)
Stage B	Patients with structural heart disease who have never had signs or symptoms of HF
B1	Patients with ECG changes (arrhythmias or conduction disorders) may present mild echocardiographic abnormalities (abnormalities of regional contractility), but global ventricular function is normal
B2	Patients with global ventricular dysfunction (decreased LV ejection fraction)
Stage C	Patients with LV dysfunction and prior or current symptoms of HF (NYHA I, II, III, and IV)
Stage D	Patients with symptoms of HF at rest, refractory to maximized medical therapy (NYHA IV) requiring specialized and intensive interventions

**Table 2:** Recommendation to treatment of CCC [6, 10, 11].

Drug groups and interventions	Indication	Recommendation	Evidence
Renin-angiotensin-aldosterone blockers	ACEi or ARB (for those intolerant to the former) in patients with LV systolic dysfunction, LVEF < 45%, and NYHA I/II/III/IV stages B1 to D	I	C
	Spironolactone or eplerenone in patients with LV systolic dysfunction, LVEF < 35% and NYHA III/IV stages B2 to D	I	B
Beta blockers	Carvedilol, bisoprolol, and metoprolol succinate in patients with LV systolic dysfunction, LVEF < 45%, and NYHA I/II/III/IV stages B2 to D	IIa	B
Diuretics	Patients with signs and symptoms of congestion (NYHA II to IV)	I	C
Hydralazine and nitrate	Patients of any ethnicity, with LV systolic dysfunction, LVEF < 45%, and NYHA II–IV stages B2 to D with contraindications or intolerance to ACEI and ARB (e.g., progressive renal failure or hyperkalemia)	I	C
	Patients with LV systolic dysfunction, LVEF < 45%, and NYHA III-IV as an addition to the use of optimized therapy stages B2 to D	IIa	C
Digitalis	Patients with LV systolic dysfunction, LVEF < 45%, and sinus rhythm or atrial fibrillation, symptomatic despite optimized therapy stages B2 to D	IIa	C
	Patients with LV systolic dysfunction, LVEF < 45%, and AF, asymptomatic, to control high heart rate	III	C
Oral anticoagulation	Atrial fibrillation, previous embolic events, mural thrombus, IPEC/FIOCRUZ score ≥ 4	I	C
Amiodarone	Patients with ventricular ectopy, asymptomatic NSVT, and left ventricular dysfunction stages B2 to D	I	B
	Patients with symptomatic SVT or not, with or without left ventricular dysfunction not treated with ICD stages B1 to D	I	C
	To reduce shocks in patients with ICD stages B1 to D	I	C
	Patients with symptomatic SVT treated with CDI stages B2 to D	IIa	C
ICD	Malignant arrhythmia, or sustained ventricular tachycardia, or those resuscitated from sudden cardiac arrest, especially with a reduced LVEF. Stages B2 to D	I	C
Resynchronization	Refractory HF, or functional class III/IV with persistent therapeutic optimization and any evidence of dyssynchrony. Sinus rhythm, QRS duration **>**120 milliseconds, and LVEF **<**35%. Stages B2 to D	IIb	C
Heart transplantation	Refractory HF, dependent on inotropic drugs and/or circulatory support and/or mechanical ventilation stages C to D	I	C
	VO2 peak ≤ 10 mL/kg/min, or if in use of beta blockers with VO2 peak = 12 mL/kg/min stages C to D	I	C
	Fibrillation or sustained refractory ventricular tachycardia stages C to D	I	C
	Functional class III/IV with persistent therapeutic optimization stages C to D	I	C
Ventricular circulatory support	Bridge to heart transplantation, destination therapy, or bridging to recovery. Stages C to D	Few evidence	Few evidence
Immunoadsorption (IA)	Based on other cardiomyopathies, without evidence of CCC yet	No evidence	No evidence
Aptamers treatment	Studies in progress	No evidence	No evidence
Specific treatment	Acute infections, independently of the mechanism of transmission (consensual indication)	I	B
	High-risk accidental contaminations (consensual indication)	I	B
	Chronic phase in children (consensual indication)	I	B
	Reactivated *Trypanosoma cruzi *infection—AIDS or other immunosuppression (consensual indication)	I	C
	Congenital infection (consensual indication)	I	B
	Organ transplantation in which either the donor or the recipient has Chagas' disease (consensual indication)	I	B
	Late, chronic phase, including patients with the indeterminate or cardiac forms of Chagas' disease (not consensual indications)	III	C
